# The CRISPR-Cas12a Platform for Accurate Genome Editing, Gene Disruption, and Efficient Transgene Integration in Human Immune Cells

**DOI:** 10.1021/acssynbio.2c00179

**Published:** 2023-02-07

**Authors:** Marina Mohr, Nkerorema Damas, Johanne Gudmand-Høyer, Katrine Zeeberg, Dominika Jedrzejczyk, Arsenios Vlassis, Martí Morera-Gómez, Sara Pereira-Schoning, Urška Puš, Anna Oliver-Almirall, Tanja Lyholm Jensen, Roland Baumgartner, Brian Tate Weinert, Ryan T. Gill, Tanya Warnecke

**Affiliations:** † Novo Nordisk Foundation Center for Biosustainability, Technical University of Denmark, Kemitorvet 220, 2800 Kongens Lyngby, Denmark; ‡ Artisan Bio, 363 Centennial Parkway, Suite 310, Louisville, Colorado 80027, United States

**Keywords:** CRISPR, MAD7, NHEJ, HDR, frameshift mutations, CAR T-cells

## Abstract

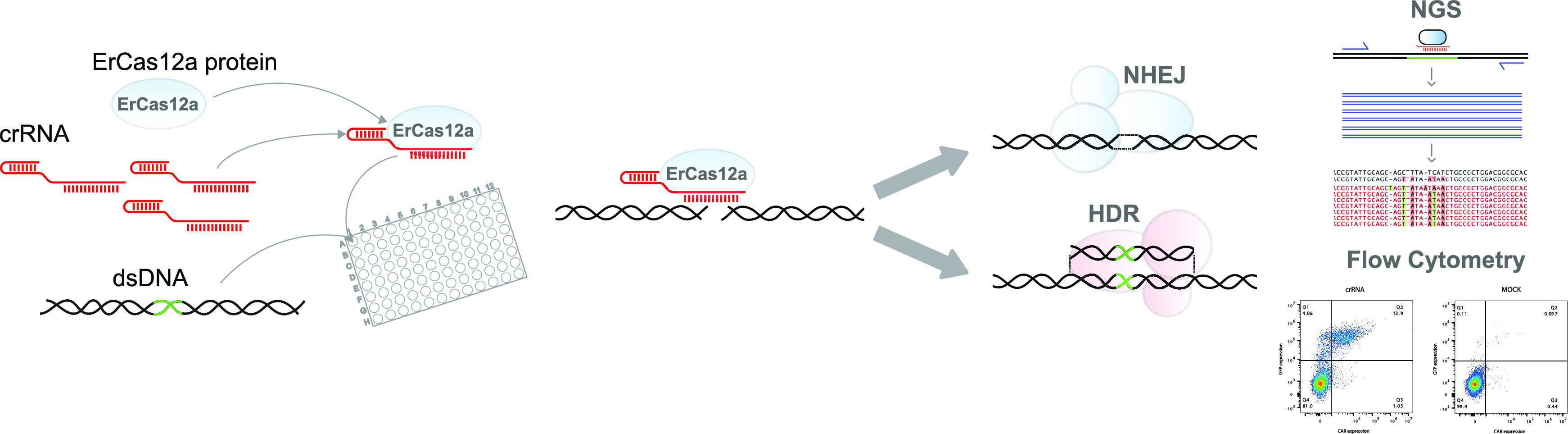

CRISPR-Cas12a nucleases have expanded the toolbox for targeted genome engineering in a broad range of organisms. Here, using a high-throughput engineering approach, we explored the potential of a novel CRISPR-MAD7 system for genome editing in human cells. We evaluated several thousand optimization conditions and demonstrated accurate genome reprogramming with modified MAD7. We identified crRNAs that allow for ≤95% non-homologous end joining (NHEJ) and 66% frameshift mutations in various genes and observed the high-cleavage fidelity of MAD7 resulting in undetectable off-target activity. We explored the dsDNA delivery efficiency of CRISPR-MAD7, and by using our optimized transfection protocol, we obtained ≤85% chimeric antigen receptor (CAR) insertions in primary T cells, thus exceeding the baseline integration efficiencies of therapeutically relevant transgenes using currently available virus-free technologies. Finally, we evaluated multiplex editing efficiency with CRISPR-MAD7 and demonstrated simultaneous ≤35% CAR transgene insertions and ≤80% gene disruption efficiencies. Both the platform and our transfection procedure are easily adaptable for further preclinical studies and could potentially be used for clinical manufacturing of CAR T cells.

## Introduction

The previous decade has seen dramatic improvements in genome engineering technologies with remarkable potential for advancing synthetic biology, applied biotechnology, and biomedical research.^1^ In the field of functional genomics, CRISPR technologies provide one of the most robust and flexible toolkits for reprogramming genetic sequences, disrupting endogenous genes, and inserting exogenous transgenes.^2^ The most widely adopted CRISPR system for creating site-specific double-strand breaks in DNA utilizes the Cas9 endonuclease (class 2 type II), which requires the tracrRNA–crRNA duplex and the presence of a short G-rich PAM sequence adjacent to the targeted sequence in the DNA.^3^ As of 2015, Cas12a (class 2 type V) nucleases have received much attention due to their (1) utilization of a single, short RNA molecule (crRNA) that reduces the complexity of the editing systems as well as the cost of synthesis; (2) ability to process crRNA arrays, which facilitates multiplexed editing strategies;^4^ (3) requirement for T-rich PAMs, which enables targeting of genomic regions unrecognized by Cas9;^5^ and, among others, (4) reduced off-target activities relative to their Cas9 counterpart.^6,7^ The most commonly used Cas12a nucleases, AsCas12a and LbCas12a, are both characterized by 5′-TTTV-3′ PAM recognition motifs, although some diversity among orthologs has been observed.^8,9^ More recently, an alternative and royalty-free CRISPR-Cas12a nuclease developed from *Eubacterium rectale* (ErCas12a), known as MAD7, was described to target a broader range of PAM sequences, namely, 5′-YTTN-3′, and has demonstrated high gene editing activities in microbial systems^10^ and adequate editing efficiencies, via non-homologous end joining (NHEJ), in eukaryotic systems including mammalian cancer cell lines.^11,12^

A major emphasis in the use of CRISPR technology for therapeutic applications is on genetic modification and redirection of human immune cells to target overexpressed antigens for treatment of tumors among other indications. The first clinical applications relied upon viral vectors for random or semi-random insertion of DNA payloads containing exogenous transgenes including chimeric antigen receptors (CARs).^13^ These produced promising results and set the stage for future applications that now include site-specific integration of CAR transgenes as well as multiplex editing of immunologically relevant genes for enhanced efficacy, including PDCD1, TIM3, LAG3, TIGIT, and CTLA4.^14^ Preclinical studies suggest that targeted integration of CAR transgenes enables regulated transgene expression and enhanced functionality of T cells^15,16^ while reducing the risk of insertional mutagenesis that may occur through random or semi-random integration, which leads to unpredictable and variable expression of the therapeutic gene.^17^ While viral-free, site-specific transgene integrations achieved by co-transfection of CRISPR-Cas9 ribonucleoproteins (RNPs) and short homology-directed repair (HDR) templates (<1000 bp) have been efficiently integrated into various genetic loci in human primary T cells,^18^ this approach has been less efficient for the integration of large, therapeutically relevant transgenes, e.g., anti-CD19 CAR, for adoptive T-cell transfer (5–15%).^18−20^ Recently, however, an optimized CRISPR-Cas9 method built on the currently available virus-free technology^18,21^ has improved the integration efficiencies of CAR transgenes into the TRAC locus, achieving delivery efficiencies of ≤68% in primary T cells.^22^ To the best of our knowledge, no comparable integration efficiencies of therapeutically relevant transgenes into the human genome using CRISPR-Cas12a have been reported.

Here, we describe the engineering and use of ErCas12a or MAD7 as a basis for a scalable, high-throughput platform for human cell engineering. First, we engineered MAD7 for optimal expression and subsequent purification, which was then used to optimize conditions for CRISPR-MAD7 RNP-driven editing in T cells using a 96-well plate format.^18^ We next demonstrated the scalability of this approach by screening several hundred crRNAs adjacent to T-rich PAM sites (5′-YTTN-3′) at eight immunologically relevant loci resulting in specific MAD7-crRNAs that enable high NHEJ editing frequencies at several immune checkpoint receptor, checkpoint phosphatase, and TCR signaling subunit genes. Next, we mined the complete dataset to inform MAD7 sequence specificity and PAM preferences and determined the off-target activity in cells, which enabled high-confidence scalability of our workflows to more advanced applications. Finally, we explored the integration of exogenous genes at the AAVS1 safe-harbor site using our optimized single-gene and multiplex transfection methods and demonstrated extraordinary transgene integration efficiencies and optimally regulated gene expressions.

## Results

### The CRISPR-MAD7 Platform for Human Genome Editing Using the Jurkat T-Cell Leukemia Cell Line

In mammalian cells, transfection of endonuclease and crRNA RNP complexes enables high-efficiency genome editing with reduced off-target editing, compared to plasmid-based systems that constitutively express the CRISPR protein and crRNA.^23,24^ To enable genome editing in human cells, a codon-optimized MAD7 sequence^25^ was flanked with either a single or quadruple nuclear localization signal (NLS) (Figure S1a). The *in-cellulo* editing activity of the modified MAD7 nuclease was determined by nucleofection of RNPs in a T-cell leukemia cell line (Jurkat), followed by amplification of the edited part of the locus, targeted next-generation sequencing for identification of the edits, and finally computational analysis of modification frequency using the CRISPResso2 algorithm.^26^

**Figure 1:**
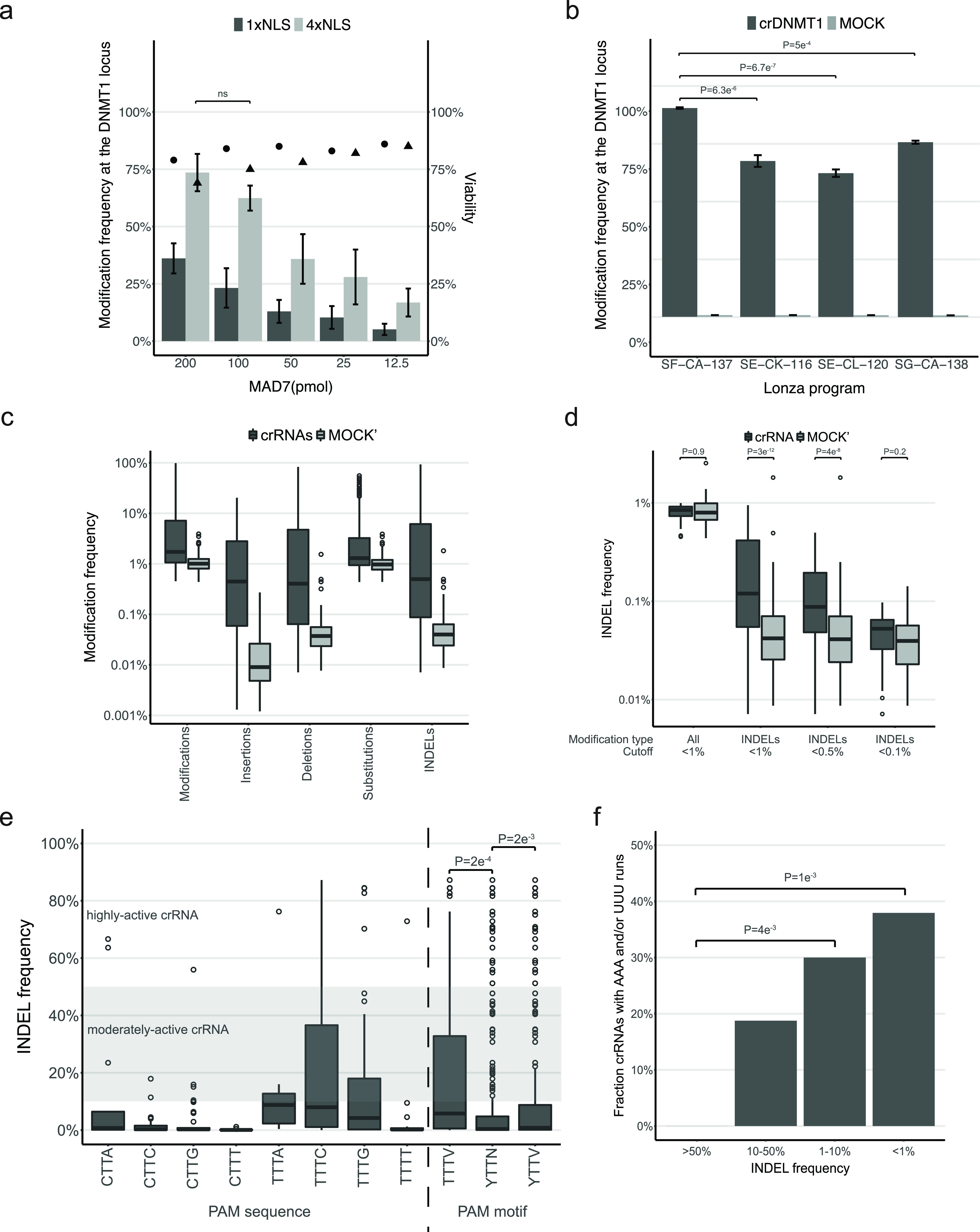
(a) MAD7 and RNP optimization. Modification frequency at the DNMT1 locus (*t* = 3; mean ± SD) and cell viability (*t* = 3; mean) of Jurkat cells as a function of single (1×) or quadruple (4×) nuclear localization signal (NLS) and MAD7-RNP amounts (pmol; constant ratio of 2:3 MAD7:crRNA) for improved in cellulo editing. Modifications were achieved using the Lonza-recommended nucleofection program SE-CL-120 for the Jurkat cell line. Dark gray bars and circles represent mean modification frequency and viability, respectively, using 1× NLS-MAD7. Light gray bars and triangles represent mean modification frequency and viability, respectively, using 4× NLS-MAD7. Modification frequency at the DNMT1 locus using 200 pmol MAD7 (constant ratio of 2:3 MAD7:crRNA) is statistically insignificant compared to 100 pmol MAD7 (two-tailed *T*-test, *P* ≥ 0.05). (b) Transfection condition optimization. Modification frequency at the DNMT1 locus (*t* = 4; mean ± SD) in the Jurkat cell line achieved by utilization of the optimized transfection conditions (Figure 1a; 100 pmol 4× NLS-MAD7) and Lonza-recommended nucleofection programs SE-CK-116 and SE-CL-120, as well as the two best nucleofection programs observed in this study, SF-CA-137 and SG-CA-138 (Figure S1b). Modification frequency at the DNMT1 locus is significantly higher using SF-CA-137 compared to other programs (two-tailed *T*-test, *P* ≥ 0.05). Dark gray and light gray bars represent mean modification frequency in treated and MOCK (crIDTneg1, IDT) samples, respectively. (c) Detection of the editing event. Modification frequency at eight different loci using 298 crRNAs (*t* = 3; mean ± SD) in the Jurkat cell line as a function of various modification types: all Modifications, Insertions, Deletions, Substitutions, or Insertions and Deletions (INDELs). Modifications were achieved using the optimized transfection conditions (Figure 1a; 100 pmol 4× NLS-MAD7) and the best-performing Lonza nucleofection program (Figure 1b; SF-CA-137). Dark gray and light gray boxplots represent mean modification frequency in treated and MOCK’ samples (no MAD7), respectively. (d) Sensitivity of assay. INDEL frequency at eight different loci using 298 crRNAs (*t* = 3; mean ± SD) in the Jurkat cell line as a function of two modification types: all Modifications ≤1%, and INDELs ≤1%, or ≤0.5%, or ≤0.1%, with significantly lower INDEL frequencies in MOCK compared to crRNA reactions at INDELs ≤1% (Fisher’s exact test; *P* = 3 × 10^–12^) and ≤0.5% (Fisher’s exact test, *P* = 4 × 10^–8^). Transfection protocol as described in (c). Dark gray and light gray boxplots represent mean INDEL frequency in treated and MOCK’ samples (no MAD7), respectively. (e) PAM motifs. INDEL frequency at eight different loci using 298 crRNAs (*t* = 3; mean ± SD) in the Jurkat cell line as a function of eight YTTN PAM combinations, and TTTV, YTTN, and YTTV PAM motifs. A gray zone on the plot represents moderately active crRNAs (10–50% INDELs), the zone above highly active crRNAs (≥50% INDELs), and the zone below active crRNAs (1–10% INDELs). INDEL frequency at the YTTV and TTTV PAM motifs is significantly higher compared to the YTTN motif (Fisher’s exact test, *P* = 2 × 10^–3^ and *P* = 2 × 10^–4^, in that order). Transfection protocol as described in (c). (f) crRNA sequence logos. Fraction of crRNAs with AAA and/or UUU runs as a function of INDEL frequency of highly active (≥50% INDELs), moderately active (10–50% INDELs), active (1–10% INDELs), and inactive (≤1% INDELs) crRNAs. Transfection protocol as described in (c). Fraction of inactive (≤1% INDELs) and active (1–10% INDELs) crRNAs containing such runs is significantly higher compared to highly active (≥50% INDELs) crRNAs (Fisher’s exact test, *P* = 1 × 10^–3^ and *P* = 4 × 10^–4^, respectively).

Firstly, using a crRNA targeting the DNMT1 locus, we compared the editing efficiency of different RNP amounts with either 1 × NLS or 4 × NLS MAD7. We observed RNP concentration-dependent modification efficiency, as evidenced by an increased fraction of modified amplicons with increased amounts of RNP complexes (Figure 1a). Notably, editing was markedly enhanced by use of 4 × NLS compared to 1 × NLS tagged MAD7, which indicates that optimization of the NLS is important to achieve maximal editing efficiency. Moreover, we observed that the fraction of modified amplicons increased along with the concentration of RNP, while the viability of cells concurrently decreased with the increasing concentration of RNP complexes (Figure 1a). In summary, the highest modification frequency and cell viability were achieved using 100 pmol 4 × NLS-MAD7 per reaction (2:3 MAD7:crRNA).

To optimize in cellulo editing activity in our model system, we tested 96 different transfection conditions—using 32 nucleofection programs in combination with three buffers—on the Lonza Nucleofector 96-well Shuttle System. The majority of the buffer-program transfection combinations resulted in suboptimal viability and modification frequency; however, our analysis revealed seven out of 96 conditions that supported substantial rates of both cell viability and editing efficiency (both ≥70%; Figure S1b). The two best transfection conditions observed in the screen, namely, SF-CA-137 and SG-CA-138, were then validated and compared to the Lonza-recommended nucleofection programs for Jurkat cell lines, namely, SE-CL-120 and SE-CK-116. We established that the optimal modification frequency and cell viability are achieved using the SF-CA-137 transfection condition (Figure 1b).

### Scalable High-Level MAD7-RNP Editing of Immunologically Relevant Genes in the Jurkat T-Cell Leukemia Cell Line

In this study, we used the Jurkat cell line as a model system to screen crRNAs for high-efficiency editing sites. The screen included 298 unique crRNAs targeting the immune checkpoint receptors PDCD1, TIM3, LAG3, TIGIT, and CTLA4; the checkpoint phosphatases PTPN6 (SHP-1) and PTPN11 (SHP-2); and the TCR signaling subunit CD247 (CD3ζ) (Table S1). The activity of the crRNAs was determined by targeted NGS and CRISPResso2 data analyses.

CRISPResso2 software reports the frequency of modifications (insertions, deletions, and substitutions) within a quantification window flanking the position of MAD7-induced cleavage in the amplicon sequence. To better understand the detection of editing events, we first compared the type of modifications detected in 230 amplicons that were sequenced in both crRNA-treated and MOCK’ samples (no MAD7). We observed relatively high modification frequencies (median 1%) in MOCK’ reactions as a result of high frequency of substitutions (Figure 1c); substitutions were detected at a median frequency of 0.96%, likely due to the errors in NGS base calling or substitutions arising during DNA amplification, while insertions and deletions were found at much lower median frequencies of 0.003 and 0.042%, respectively. Thus, we used the frequency of both insertions and deletions (INDEL) to quantify the editing activity of the CRISPR-MAD7 system. Moreover, we observed that low INDEL frequencies in MOCK’ reactions enabled the sensitive detection of editing events at a significantly greater fraction of sites (*P* = 3e^–12^). Analysis of crRNAs with low INDEL frequencies showed statistically significant editing in crRNA-treated samples compared to MOCK’ samples at INDEL frequencies as low as 0.5% (*P* = 4e^–8^; Figure 1d). This indicates the sensitivity of the assay to detect modifications in the sub-1% range. The low INDEL frequency in MOCK’ amplicons also suggests that the controls are largely superfluous when screening for active crRNAs with ≥10% INDEL frequency and can be reserved for validation experiments.

Since MAD7 is described to target a wide range of PAM sequences, we screened crRNAs adjacent to all YTTN PAM variants, which enabled the analysis of MAD7 specificity in mammalian cells. Our data indicate that MAD7 edited sites with all eight combinations of YTTN PAM; however, editing was significantly higher at the YTTV and TTTV consensus sequences (*P* = 2 × 10^–3^ and *P* = 2 × 10^–4^, respectively). While the majority of highly active crRNAs (≥50% INDEL frequency) were found at sites with YTTV and TTTV PAMs, moderately active crRNAs (≥10% INDEL frequency) were found to target every PAM sequence with the exception of CTTT. This indicates that MAD7 can edit a wide range of target PAMs, albeit at reduced frequencies (Figure 1e).

Given the large number of crRNAs analyzed, we next determined if the targeted DNA sequence biases editing efficiency. Sequence logos were made to compare the DNA–complementary crRNA sequences of inactive (≤1% INDELs), active (1–10% INDELs), moderately active (10–50% INDELs), and highly active (≥50% INDELs) crRNAs (Figure S2a). While there were no strong biases for ribonucleotides at specific positions, guanine appeared to be overrepresented and uracil underrepresented on moderately active and highly active crRNAs. Next, we examined the frequency of ribonucleotide bases within the same four classes of crRNAs (Figure S2b), and the analysis confirmed significant enrichment of guanine and depletion of uracil on highly active crRNAs. Moreover, the data showed that nearly 40% of inactive crRNAs had runs of three or more adenine or uracil ribonucleotides, while none of the highly active and ≤20% of moderately active crRNAs contained such runs (Figure 1f). While these sequence features cannot determine crRNA activity, they may be useful for selecting putative high-activity crRNAs during initial rounds of screening and could reduce the overall cost of identifying crRNAs for various genes of interest or increase the overall scale of genes studied.

### Validation of crRNAs for Gene Editing and Disruption of Immunologically Relevant Genes Using the Jurkat T-Cell Leukemia Cell Line

**Figure 2:**
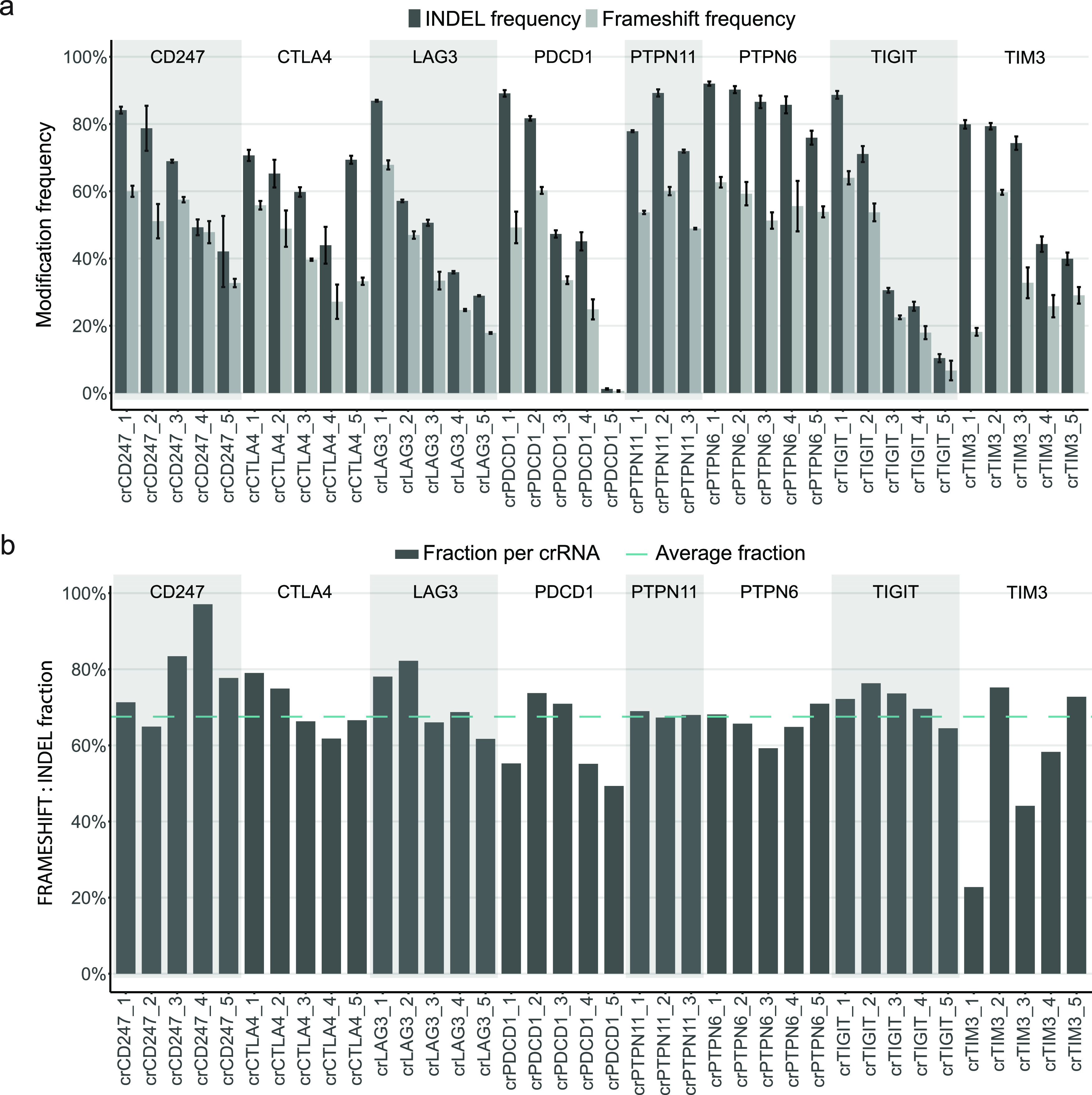
(a) INDEL and frameshift frequencies. INDEL (dark gray bars) and frameshift (light gray bars) frequencies (*t* = 3; mean ± SD) in the Jurkat cell line as a function of 38 highly active crRNAs. Alternating gray and white zones on the plot represent groups of three to five highly active crRNAs per locus. Transfection protocol as described in Figure 1c. (b) Fraction of frameshift to INDEL frequency. Fraction of frameshift to INDEL frequency (dark gray bars) in the Jurkat cell line as a function of 38 high-efficiency crRNAs. Average fraction of INDELs leading to frameshifts (dashed line) is approximately 66%. Alternating gray and white zones on the plot represent groups of three to five highly active crRNAs per locus. Transfection protocol as described in Figure 1c.

We next validated high-efficiency crRNAs identified in our initial analysis by assaying INDEL frequency for the top three or five crRNAs for each of the selected immunologically relevant genes (Figure 2a). In the validation experiment, the INDEL frequency was significantly correlated with the measurements from the initial screen, highlighting the reproducibility of the INDEL assay (Figure S3). Using the CRISPResso2 software, we then estimated the degree of open reading frame (ORF) disruption for each of the validated crRNAs (Figure 2a). In addition, for four highly active crRNAs targeting three different exons at the PDCD1 locus, we measured the surface expression of the PDCD1 protein by flow cytometry 4, 7, and 11 days post-transfection. The data revealed that the protein surface expression after transfection with crPDCD1_1, a crRNA targeting the PDCD1 gene at the extracellular domain of the protein, was as low as 4% 4 days post-transfection and remained at this level even at day 11 post-transfection. The surface expression after transfection with the remaining three crRNAs was significantly higher, 19% and 46% after transfection with crPDCD1_2, and both crPDCD1_3 and crPDCD1_4, respectively. It is important to emphasize that induced alterations in the proximity of the transmembrane domain (crPDCD1_2) or the cytoplasmic domain (crPDCD1_3 and crPDCD1_4) might not be reflected in the expression measurements of the surface protein (Figure S4). This is in line with the ORF data analysis, which showed that for most of the crRNAs including the highly active crPDCD1s, the predicted number of INDELs leading to frameshifts was similar to what we would expect from an unbiased DNA repair process, with frameshifts in two-thirds of the edited loci (Figure 2b). However, several of the crRNAs had a markedly different degree of ORF disruption; crCD247_4 resulted in frameshifts with 97% frequency, while crTIM3_1 and crTIM3_3 resulted in frameshifts with 23% and 44% frequencies, respectively (Figure 2b). The analysis of repair products indicates that in the case of crTIM3_1, and to some extent crTIM3_3, the bias arose from directly repeated sequences at the DNA cleavage site, which possibly promoted microhomology-mediated end joining (MMEJ) repair following DNA cleavage. These data help inform the selection of crRNAs for gene KO since some crRNAs, such as crTIM3_1, have a much lower frequency of gene disruption than would be predicted based on the frequency of INDEL formation.

**Figure 3:**
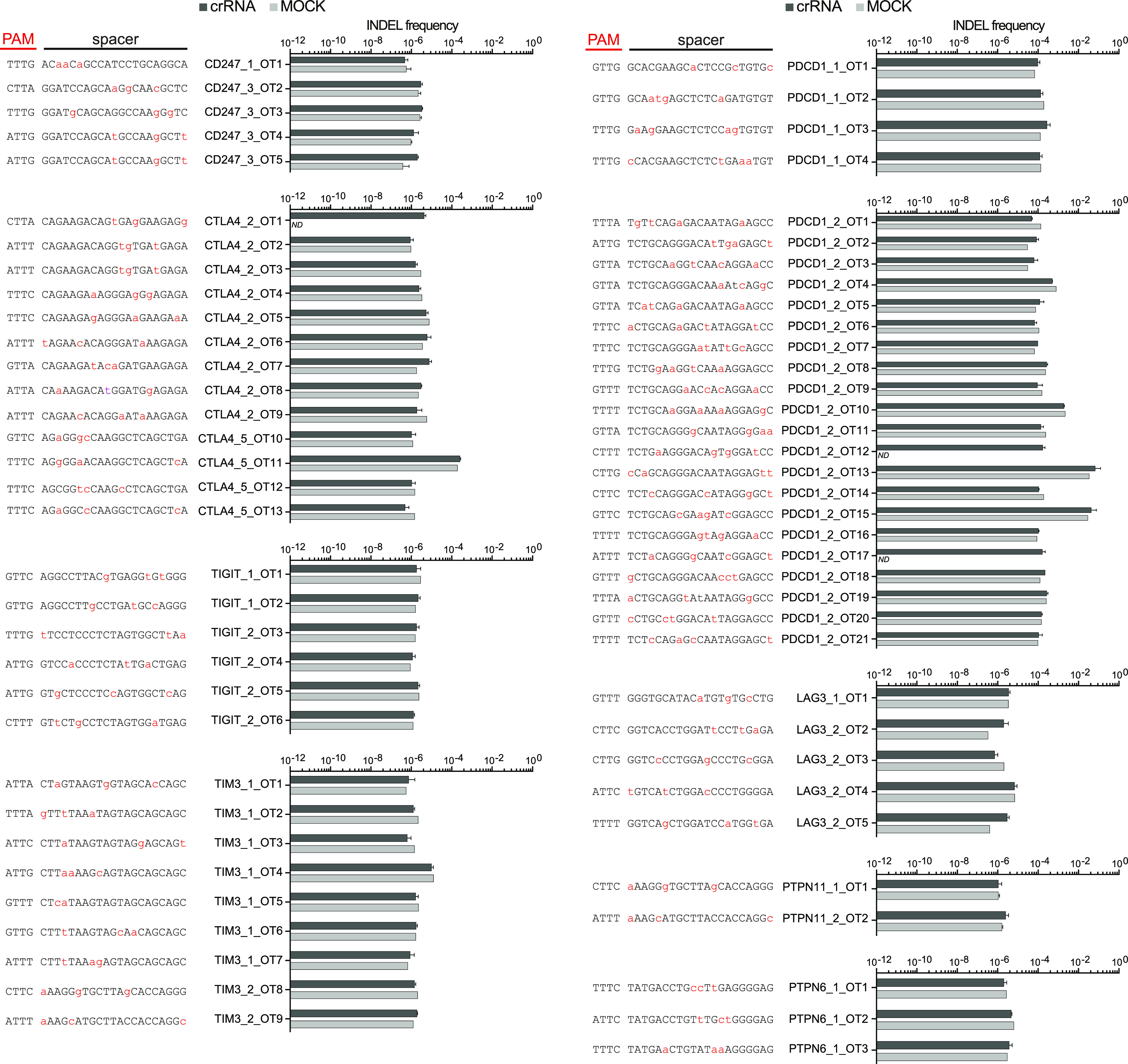
Off-target analysis. INDEL frequency of MAD7 (*t* = 3; mean ± SD) in the Jurkat cell line at predicted off-target sites analyzed by targeted deep sequencing. For crPDCD1, the INDEL frequency was analyzed at the putative off-target editing sites with ≤4 mismatches between the crRNA and target DNA sequence, and with ≤3 mismatches on the remaining crRNAs. PAM sequences and spacer sequences with mismatches marked in red are displayed next to their respective measured INDEL frequencies. No significant INDEL frequency at any of the off-target sites was detected (two-tailed *T*-test, *P* ≥ 0.05).

Another consideration for selecting crRNAs is the potential for off-target cleavage events. We analyzed the list of validated crRNAs using the Cas-OFFinder software^27^ to predict potential off-target editing sites in the genome with up to four mismatches between the crRNA and the target DNA sequence. Using the Bioconductor R packages,^28−31^ we then matched the predicted off-target sites with the human gene database and extracted those sites that targeted exons and introns within the genes. Afterward, we examined the degree of editing activity at these sites by targeted next-generation sequencing, more specifically at 25 predicted off-target sites for the top-two PDCD1 crRNAs, i.e., crPDCD1_1 and crPDCD1_2. The analysis revealed low-level off-target activity at crPDCD1_2_13 and crPDCD1_2_15 sites; however, INDEL formation at these two sites was statistically insignificant compared to MOCK samples (non-targeting crRNA) (*P* ≥ 0.05; Figure 3). We further assayed the INDEL frequency at 43 putative off-target sites with up to three mismatches between crRNA and target DNA sequences for the top-two crRNAs targeting seven remaining genes (i.e., TIM3, LAG3, TIGIT, CTLA4, PTPN6, PTPN11, and CD247). The analysis revealed no detectable activity at any of the putative off-target sites (Figure 3), which reinforces the high cleavage fidelity of MAD7.

### Transgene Insertion in the Jurkat T-Cell Leukemia Cell Line with the CRISPR-MAD7 Platform

Insertion of exogenous transgenes is an important aspect of mammalian cell engineering. Gene insertion with CRISPR-Cas is achieved by HDR of CRISPR-induced DNA breaks using HDR-donor templates to integrate exogenous genetic sequences into targeted DNA loci. Several studies indicate that HDR templates, composed of linear double-stranded DNA, provide the most robust and efficient method of transgene insertion using CRISPR-Cas genome editing systems.^18,22^

**Figure 4:**
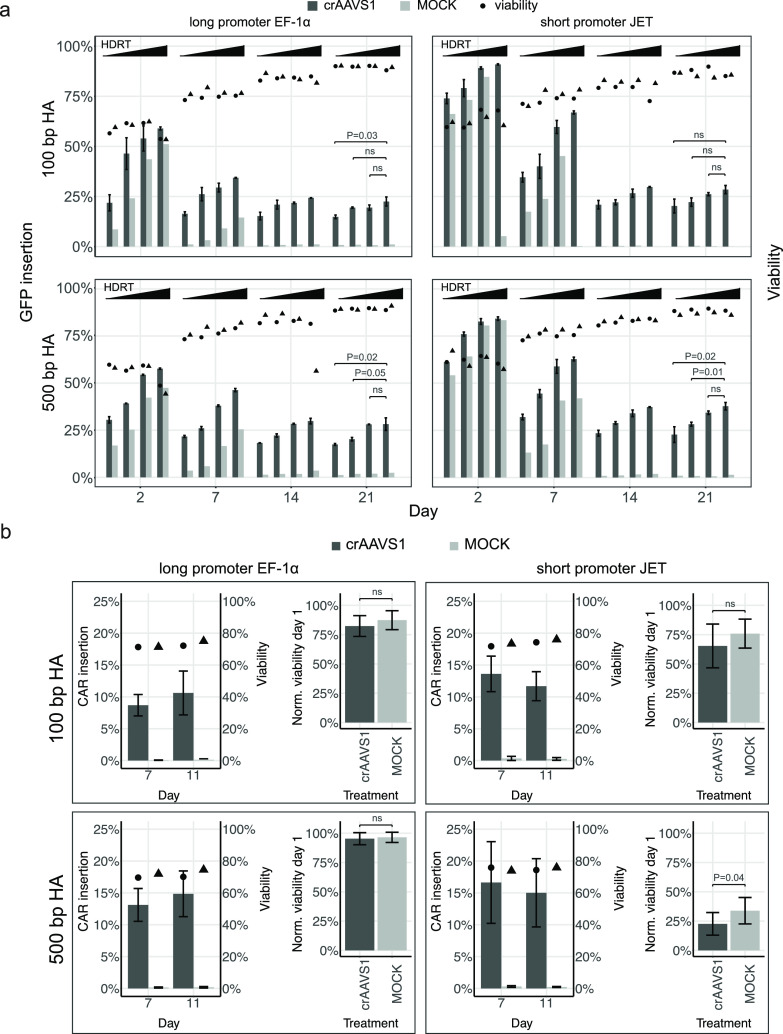
(a) Transgene insertion in Jurkat cells. Non-normalized cell viability (circles and triangles; *t* = 3; mean) and GFP insertion efficiency at AAVS1 (*t* = 3; mean ± SD) in the Jurkat cell line measured at days 2, 7, 14, and 21 post-transfection as a function of HDRT amount. Top panels display GFP insertion efficiencies using HDRT flanked with short HAs (100 bp HAs), and bottom panels HDRT flanked with long HAs (500 bp HAs). Left panels display GFP insertion efficiencies using HDRT containing EF-1α promoter (long), and right panels HDRT containing JET promoter (short). GFP insertion efficiency is statistically significant only between JET-500 bp HA and EF-1α-100 bp HA (*P* = 0.021). No significant differences are found among other promoter-HA length combinations (two-tailed *T*-test, *P* ≥ 0.05). Amount of HDRT, represented by the gradient above the bars, increases from 0.125, 0.25, 0.5 to 1 μg. Statistical significance of increasing HDRT amounts on GFP insertion efficiencies at day 21 (no transient GFP expression) is depicted above the bars (two-tailed *T*-test, *P* ≥ 0.05). Dark gray and light gray bars represent mean GFP insertion frequency in treated and MOCK (crIDTneg1) samples, in that order; black circles and triangles represent mean non-normalized cell viability in treated and MOCK (crIDTneg1) samples, respectively. Transfection protocol as described in Figure 1c. (b) Transgene insertion in primary T-cells. Non-normalized cell viability (circles and triangles; *t* = 3; mean) and CAR insertion efficiency at AAVS1 (*N* = 3; *t* = 3; mean ± SD) in primary Pan-T cells measured at days 7 and 11 post-transfection. Normalized cell viability (*N* = 3; *t* = 3; mean ± SD) at 24 h post-transfection (two-tailed *T*-test, *P* ≥ 0.05). Normalized cell viabilities at 24 h post-transfection are significantly different between EF-1α-100 bp HA and EF-1α-500 bp HA (*P* = 0.021), EF-1α-100 bp HA and JET-100 bp HA (*P* = 0.028), and EF-1α-100 bp HA and JET-500 bp HA (*P* = 1.9 × 10^–4^). No significant differences are found among other promoter–HA length combinations (two-tailed *T*-test, *P* ≥ 0.05). Individual panels display CAR insertion efficiencies using the HDRT structure as described in Figure 4a. CAR insertion efficiency is statistically significant only between EF-1α-100 bp HA and EF-1α-500 bp HA (*P* = 0.049). No significant differences are found among any other promoter–HA length combinations (two-tailed *T*-test, *P* ≥ 0.05). The amount per reaction of HDRT, MAD7-RNP, and PGA was 1 μg, 100:150 pmol MAD7:crRNA, and 160 μg, in that order. Nucleofection program P3-EH-115 for transfection of primary T cells was used. *N* represents the number of biological replicas, and *t* the number of technical replicas per *N*. Dark gray and light gray bars represent mean insertion frequency and mean normalized cell viability (relative to UNMOCK) in treated and MOCK (crIDTneg1) samples, in that order; black circles and triangles represent mean non-normalized cell viability in treated and MOCK (crIDTneg1) samples, respectively.

Here, we used the Jurkat cell line to evaluate the transgene insertion and expression efficiency using CRISPR-MAD7. We used a highly active crRNA targeting the AAVS1 safe-harbor locus (Figure S5; Vlassis et al. *under review*) and eight different HDR-repair templates flanked with symmetric homology arms (HAs) of 500 base pairs (bp) in the amount of 0.5 μg reaction^–1^. The HDR inserts consisted of eight promoters differing in size and promoter strength to drive GFP expression (Figure S6). When the transient GFP expression diminished at day 14 post-transfection, we observed comparable insertion efficiencies with stable GFP expressions of up to 30% using four out of eight promoters, that is, JET (195 bp), PGK (511 bp), EF-1α (1195 bp), and CAG (1723 bp) (Figure S6), suggesting that the insert size had not affected the integration efficiency at AAVS1 in the Jurkat cell line. Subsequently, keeping the RNP amounts per reaction constant (100:150 pmol MAD7:crRNA), we evaluated the effect of various HA lengths (100 vs 500 bp) and HDR template amounts (0.125, 0.25, 0.5, and 1 μg) on the insertion efficiency using two selected promoters, namely, JET and EF-1α. We observed up to 30% higher integration efficiency with HDR templates flanked with HA of 500 compared to 100 bp. Moreover, the data showed improved insertion efficiencies with increasing amounts of HDR templates flanked with either 100 or 500 bp HA but at the same time somewhat reduced cell viability (Figure 4a).

### Transgene Insertion and Multiplex Genome Editing in Primary T Cells with the CRISPR-MAD7 Platform

To assess the potential of CRISPR-MAD7 as an editing platform for ex vivo cell therapies, we tested its performance in human primary cells. Using Pan-T cells isolated from the peripheral blood from three donors and the best procedure from the experiments above, i.e., 100:150 pmol MAD7:crRNA together with the 1 μg HDR template, in combination with 160 μg of poly-l-glutamic acid (PGA)^21^ per reaction, we evaluated the integration efficiency of a clinically relevant CAR transgene containing the JET or EF-1α promoter flanked with HA of 100 or 500 bp and a bovine growth hormone-derived polyadenylation sequence. We used an anti-CD19 CAR with fully human variable regions (Hu19CAR), CD8α hinge and transmembrane domains, a CD28 costimulatory domain, and a CD3ζ activation domain.^32^ We observed moderate insertion efficiency at AAVS1 but a stable CAR expression of up to 14% and 16% using HDR templates flanked with 100 and 500 bp HA, respectively. However, the normalized cell viability (normalized to MOCK) measured 24 h post-transfection ranged from 23% with JET-500-CAR, 65% with JET-100-CAR, 82% with EF-1α-100-CAR, to 95% with EF-1α-500-CAR. Mean cell viabilities (non-normalized) measured at day 7 and day 11 post-transfection exceeded 70% in all treatments (Figure 4b). It is important to emphasize that both CAR insertion efficiency and normalized cell viability significantly increased in the samples treated with PGA compared to the treatment without PGA (*P* ≤ 0.05; Figure S7).

**Figure 5:**
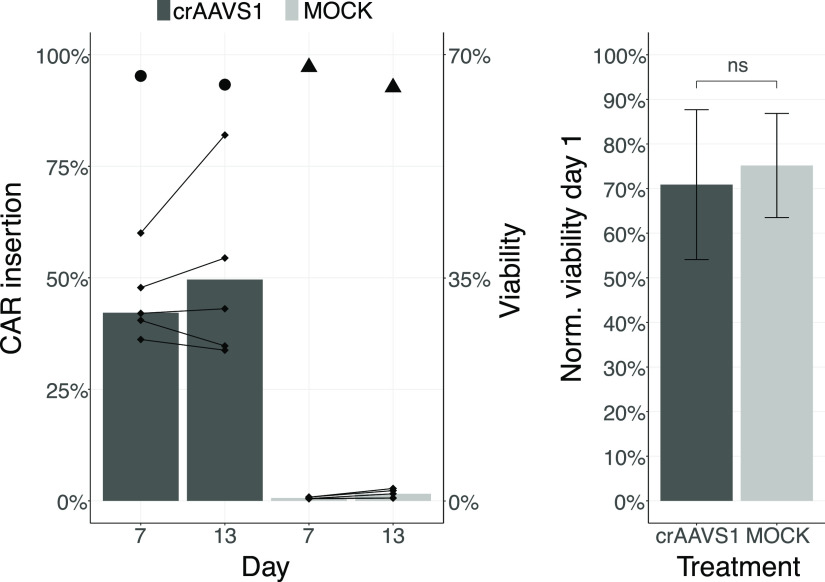
Optimized procedure for transgene insertion in primary T cells. Non-normalized cell viability (circles and triangles; *N* = 5; *t* = 3; mean) and CAR insertion efficiency at AAVS1 (*N* = 5; *t* = 3; mean) in primary Pan-T cells measured at days 7 and 13 post-transfection. Black diamonds represent mean CAR insertion efficiency of three technical replicas per donor at days 7 and 13; paired samples are linked with connecting lines. Normalized cell viability (*N* = 5; *t* = 3; mean ± SD) at 24 h post-transfection (two-tailed *T*-test, *P* ≥ 0.05). The amounts per reaction of HDRT, MAD7-RNP, PGA, and concentration of M3814 was 1.5 μg, 50:75 pmol MAD7:crRNA, 160 μg, and 2 μM, in that order. Nucleofection program P3-EH-115 for transfection of primary T cells was used. *N* represents the number of biological replicas, and *t* the number of technical replicas per *N*. Dark gray and light gray bars represent the mean insertion frequency and mean normalized cell viability (relative to UNMOCK) in treated and MOCK (crIDTneg1) samples, in that order; black circles and triangles represent mean non-normalized cell viability in treated and MOCK (crIDTneg1) samples, respectively.

We then re-evaluated multiple parameters of our procedure to further optimize primary T-cell post-transfection viability and CAR insertion efficiencies at AAVS1. Using Pan-T cells isolated from the blood from two donors, we systematically tested the effect of RNP amounts (with 160 μg of PGA) and EF-1α-500-CAR HDRT amounts on CAR insertion efficiency and cell viability (Figure S8). We observed that by reducing the RNP amount to 50:75 pmol MAD7:crRNA while increasing the HDRT amount to 1.5 μg reaction^–1^, this resulted in improved CAR insertion efficiencies without substantially affecting cell viability (Figure S8). In addition, we observed that by using the abovementioned transfection conditions in combination with the cell recovery in a post-transfection cultivation medium pretreated with 2 μM M3814, this resulted in nearly five-times more efficient CAR insertion (Figure 5; patent application US63/315483) compared to the protocol above (see Figure 4b). In summary, utilizing our optimized CRISPR-MAD7 transfection procedure resulted in a CAR insertion efficiency of up to 85% 13 days post-transfection (mean of 50% across five donors), together with the normalized cell viability (normalized to MOCK) as high as 62% 24 h post-transfection (mean 52%). Mean cell viabilities (non-normalized) measured at days 7 and 13 post-transfection surpassed 90% (Figure 5).

**Figure 6:**
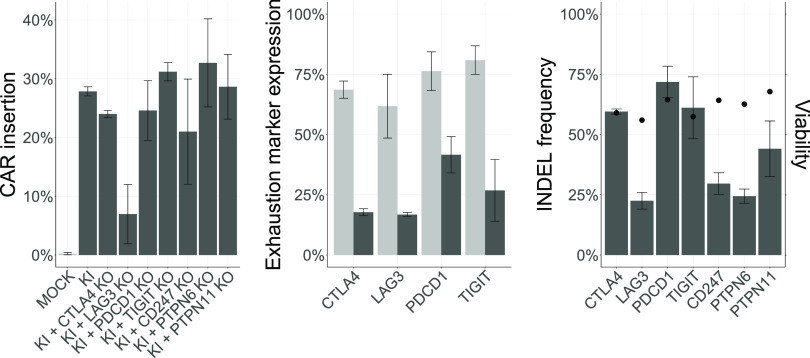
Multiplex genome editing in primary T cells. CAR insertion efficiency at AAVS1 (left panel); surface expression of CTLA4, LAG3, PDCD1, and TIGIT markers (middle panel); and INDEL frequency at CD247, CTLA4, LAG3, PDCD1, PTPN11, PTPN6, TIGIT, and TRAC loci (right panel) in double edited primary T cells measured at day 7 post-transfection (*N* = 1; *t* = 2–3; mean ± SD). MOCK—modified control; KI—CAR insertion at AAVS1; KI + CTLA4 KO—CAR insertion at AAVS1 and CTLA4 gene disruption with crCTLA4_2; KI + LAG3 KO—CAR insertion at AAVS1 and LAG3 gene disruption with crLAG3_1; KI + PDCD1 KO—CAR insertion at AAVS1 and PDCD1 gene disruption with crPDCD1_1; KI + TIGIT KO—CAR insertion at AAVS1 and TIGIT gene disruption with crTIGIT_1; KI + CD247 KO—CAR insertion at AAVS1 and CD247 gene disruption with crCD247_1; KI + PTPN6 KO—CAR insertion at AAVS1 and PTPN6 gene disruption with crPTPN6_1; and KI + PTPN11 KO—CAR insertion at AAVS1 and PTPN11 gene disruption with crPTPN11_2. Non-normalized cell viability (*N* = 1; *t* = 2–3; mean) was measured at day 7 post-transfection. The amounts per reaction of HDRT, MAD7-RNP-KI, MAD7-RNP-KO, PGA, and concentration of M3814 was 1.5 μg, 50:75 pmol MAD7:crAAVS1, 50:75 pmol MAD7:crRNA, 160 μg RNP^–1^, and 2 μM, respectively. Nucleofection program P3-EH-115 for transfection of primary T cells was used. *N* represents the number of biological replicas, and *t* the number of technical replicas per *N*. Dark gray bars represent mean insertion frequency and mean normalized exhaustion marker expression (relative to MOCK). Light gray bars represent mean insertion efficiency and mean exhaustion marker expression in the KI treatment. Black circles represent non-normalized cell viability.

The next generation of allogeneic T-cell therapies will involve multiplex genome editing to enhance the specificity, stealth, and persistence of the engineered T cells in vivo.^33^ Here, we evaluated the efficiency of multiplex genome editing with the CRISPR-MAD7 system. We used Pan-T cells isolated from human peripheral blood (one donor) and a modified procedure for transgene insertion from Figure 5: that is, 50:75 pmol MAD7:crRNA (160 μg PGA) targeting AAVS1 for CAR insertion (knock-in, KI) and 50:75 pmol MAD7:crRNA (160 μg PGA) targeting either CTLA4, LAG3, PDCD1, TIGIT, CD247, PTPN6, or PTPN11 (Figure 2) for gene disruption (knockout, KO), together with 1.5 μg of HDRT and 2 μM M3814. We observed similar CAR insertion efficiencies of approximately 30% across all treatments, except for the double-edited LAG3 treatment (Figure 6, left panel). Moreover, we observed a significantly reduced expression of surface proteins CTLA4, LAG3, PDCD1, and TIGIT relative to their respective MOCK (Figure 6, middle panel); these induced alterations were further confirmed by the INDEL assay (Figure 6, right panel). Finally, while the expression of intracellular proteins CD247, PTPN6, and PTPN11 was not measured in this study, gene modification frequencies were evaluated with the INDEL assay, which revealed INDEL frequencies of up to 30% at the CD247 and PTPN6 loci and up to 50% at the PTPN11 locus (Figure 6, right panel). The non-normalized viability of double-edited cells at day 7 post-transfection exceeded 55% across all treatments, with cell viabilities of 59, 56, 64, 57, 64, 63, and 68% in the CTLA4, LAG3, PDCD1, TIGIT, CD247, PTPN6, and PTPN11 treatments, respectively (Figure 6, right panel).

## Discussion

Non-viral reprogramming of genetic sequences has become an attractive approach for clinical manufacturing of CAR T cells as it circumvents the need of a recombinant adeno-associated virus for HDR donor template delivery,^15^ which both is costly and adds significant complexity to the overall manufacturing process. Several preclinical studies have reported successful and efficient site-specific transgene integrations by transfection of RNPs and HDR donor templates using the CRISPR-Cas9 system.^18−20,22^ While the CRISPR-Cas12a systems have shown great potential as an alternative gene editing tool to Cas9, their use has not been implemented as readily as anticipated, primarily due to the relatively low efficiency for human genome editing.^34^ Therefore, if the efficiency of CRISPR-Cas12a is optimized, these two systems can be of use for human genome editing in different genomic contexts, with Cas9 being used for editing of GC-rich and Cas12a for editing of AT-rich regions. In this study, we showed that both NHEJ editing and HDR efficiency of CRISPR-Cas12a as well as cell viability can be rapidly optimized using a high-throughput engineering approach, consequently leading to similar or even improved editing efficiencies compared to those of previously published virus-free approaches.

In this study, we explored the prospective of an engineered and optimized CRISPR-ErCas12a (MAD7) system as a gene editing tool for human genome engineering and showed that a modified 4× NLS-tagged MAD7 can be used for successful reprogramming of genetic sequences in human cells. Firstly, using our model system, we demonstrated that the optimized transfection conditions, i.e., RNP amount and nucleofection program, result in optimal cell viability and higher editing frequencies at the DNMT1 locus than previously reported for other CRISPR-Cas12a systems.^35,36^ Secondly, in the high-throughput crRNA screen, we identified highly active crRNAs, characterized by the enrichment of guanine and depletion of uracil, that allow for editing frequencies of up to 95% in addition to median 66% ORF shifts of various immune checkpoint receptors (PDCD1, TIM3, LAG3, TIGIT, CTLA4), checkpoint phosphatases (SHP-1 and SHP-2), and a TCR signaling subunit CD247 (CD3ζ). By comparison, similar NHEJ editing efficiencies at the PDCD1 locus have been achieved using AsCas12a Ultra,^46^ while merely 20% PDCD1 disruptions have been reported for Cas9.^47^ Furthermore, the crRNA screen in this study confirmed that the modified MAD7 nuclease edits sites with YTTN PAM;^11,12^ however, editing rates were significantly higher at the YTTV and TTTV consensus sequences. Finally, our data demonstrated high cleavage fidelity of the modified MAD7, which resulted in undetectable off-target activity at the predicted sites in the genome when guided by our highly active crRNAs; however, future studies should include in-depth analysis of off-target activity, as has been previously done for other CRISPR-Cas systems.^37,38^ In summary, we demonstrate that the CRISPR-MAD7 system provides a convenient platform for targeting various genomic loci with high accuracy, efficiency, and ease.

The majority of recent CAR T clinical trials utilize autologous T cells; however, their progress may be hindered by the poor quantity and quality of T cells together with the considerable resources needed for manufacturing of autologous T-cell products.^39^ Here, we explored the integration efficiency of transgenes as well as post-transfection cell viabilities using the CRISPR-MAD7 platform. Firstly, we designed eight homologous directed repair templates that consisted of promoters differing in size and strength to drive GFP expression and achieved up to 30% stable expression with JET, PGK, EF-1α, and CAG promoters at the AAVS1 safe-harbor site in the Jurkat cell line. It is important to note that the integration efficiencies of the fluorescent tag were 30% higher with the cassettes flanked with long (500 bp) compared to those flanked with short (100 bp) HAs. Next, we demonstrated a positive effect of the increasing amount of transfected dsDNA templates on the insertion efficiencies albeit at the cost of reduced viability, as also shown in previous studies.^18,22^ While loss of cell viability due to toxicity of large dsDNA donor templates is a major barrier to effective virus-free genome editing, a recent study showed that transfection of polymer-stabilized RNP particles and dsDNA template leads to reduced toxicity of the DNA.^21^ Therefore, to mitigate cell death, we tested our best transfection method (co-transfection of 1 μg of HDRT with symmetric 500 bp HA, and 100:150 pmol MAD7:crRNA) in combination with the anionic nanoparticle PGA. We achieved moderate but stable CAR insertion rates of up to 16%, with normalized cell viabilities ranging from 23 to 95% 24 h post-transfection. It is important to note that non-normalized cell viabilities steadily increased over time in all treatments, reaching 72% day 7 post-transfection and remaining at this level also at day 11. Finally, we observed that both CAR insertion efficiency and viability significantly increased in the treatments with PGA compared to the treatment without PGA, which confirms the positive effect of the anionic particle on both parameters as previously reported.^21,22^ It should also be noted that identifying optimal amounts of endonuclease, crRNA, and HDRT, along with the ratios among them, is essential to achieving the best performance of virus-free CRISPR technologies. This is especially important if any changes to the existing transfection protocols have been made (e.g., size of HDRT, promoter size, location of transgene insertion, or type of nuclease).

Next, we re-evaluated several parameters of the procedure to further optimize primary T-cell viability and CAR insertion efficiencies into the AAVS1 safe-harbor site. First, we observed that by reducing the amount of RNPs to 50:75 pmol MAD7:crRNA while further increasing the amount of dsDNA to 1.5 μg in combination with PGA, we achieved improved insertion efficiencies without significantly affecting cell viability. In addition, we observed a substantial increase in CAR integration efficiency in primary T cells treated with the M3814 inhibitor of DNA-dependent protein kinase (DNA-PK) post-transfection. Since DNA-PK is a key driver of the NHEJ double-strand break pathway,^40^ inhibition of its activity in combination with the optimized transfection method described above resulted in CAR insertion efficiencies of up to 85% 13 days post-transfection together with the normalized mean cell viability as high as 62% 24 h post-transfection. Once again, non-normalized cell viabilities increased over time, exceeding 90% at days 7 and 13 post-transfection. Thus, the two pharmacological interventions synergistically enhanced transgene integration efficiencies as well as cell viabilities and exceeded the baseline insertion efficiencies of a CAR transgene achieved with previously published virus-free approaches using CRISPR-Cas9 (≤68%).^18−22^

In the current study, we also explored the efficiency of the CRISPR-MAD7 system for simultaneous disruption of an immunologically relevant genomic locus (CTLA4, LAG3, PDCD1, TIGIT, CD247, PTPN6, or PTPN11) and integration of a CAR transgene at a safe-harbor site. We demonstrated efficient multiplex genome editing of CRISPR-MAD7, achieving ≤35% CAR integration efficiencies together with ≤80% gene disruption or NHEJ editing efficiencies in T cells and non-normalized cell viabilities of ≤70% 7 days post-transfection. However, further optimization of the transfection protocol is needed to improve the multiplex genome editing efficiency of the system. In conclusion, we show that utilizing a high-throughput engineering approach resulted in the establishment of a CRISPR-MAD7-optimized transfection procedure, leading to extraordinary integration and optimally regulated CAR expression as well as high T-cell post-transfection viabilities. Both the platform and the procedure are easily adaptable for further preclinical studies and could potentially be scalable to suit clinical purposes in an allogeneic or autologous setting.

## Methods

### Cell Line Culture

The Jurkat human T-cell leukemia cell line (Leibniz Institute DSMZ-German Collection of Microorganisms and Cell Cultures GmbH (ACC 282)) was grown in RPMI 1640 medium (Thermo Fisher Scientific) with 10% heat-inactivated fetal bovine serum (FBS) (Thermo Fisher Scientific) supplemented with a 1% penicillin–streptomycin antibiotic mix (Thermo Fisher Scientific). Cells were cultured at 37 °C in 5% CO_2_ incubators and maintained at a density of 0.5–1.5 × 10^6^ cells mL^–1^; 24 h before transfection, the cells were passaged at 0.1 × 10^6^ cell mL^–1^. A cell culture media supernatant was periodically tested for mycoplasma contamination using the MycoAlert PLUS Mycoplasma Detection Kit (Lonza).

### Primary T-Cell Isolation and Culture

This research was performed in accordance with the Declaration of Helsinki. Human peripheral blood was obtained from healthy adults after obtaining informed consent (Technical University of Denmark-Rigshospitalet National Hospital approval BC-40). T cells were isolated from the PBMC population by immune-magnetic negative selection using the EasySep Human T Cell Isolation Kit (STEMCELL Technologies). After isolation, T cells were activated in 25 μL ImmunoCult Human CD3/CD28/CD2 T-Cell Activator (STEMCELL Technologies) per 1 mL ImmunoCult-XF T Cell Expansion Medium (STEMCELL Technologies) containing 12.5 ng mL^–1^ of Human Recombinant IL-2, 5 ng mL^–1^ of IL-7, and 5 ng mL^–1^ of IL-15 (STEMCELL Technologies) and seeded at 1.0 × 10^6^ cells mL^–1^ until transfection 48 h later. The cells were maintained at 37 °C in 5% CO_2_ incubators.

### Design of Crispr RNAs

crRNAs were designed using the Benchling CRISPR Guide RNA design tool (https://benchling.com). Briefly, the software takes a target species, Ensembl transcript ID, and a base editor PAM variant as an input. Selecting the exon sequences, the software extracts the region around splice sites based on the specified window. Afterward, the pattern of N_20_-NTTN also matched the reverse strand of the sequence. Finally, matched patterns are assigned off-target scores (lowest 0, highest 100) as well as on-target scores; however, due to lack of data, the on-target scores are currently not available for Cas12a PAMs.

### Nuclease Expression and Purification

MAD7 nuclease expression and purification followed Jedrzejczyk et al. (2022).^48^

### RNP Formulation

RNP complexes were generated by incubating relevant crRNAs with MAD7 in the molar ratio of 2:3 MAD7:crRNA for 15 min at RT immediately before transfection. For Jurkat experiments, the RNP complexes were generated by mixing the specific crRNA (150 pmol), MAD7 (100 pmol), and nuclease-free water up to 5 μL, unless otherwise stated. For T-cell experiments, 1.6 μL of an aqueous solution of 15–50 kDa PGA (100 μg μL^–1^, Alamanda Polymers; modified from Nguyen et al. [^21^]) was added to crRNAs, followed by the addition of MAD7. For multiple editing T-cell experiments, the RNP complexes for KO and KI were prepared separately and as described above, followed by the addition of nuclease-free water to the final RNP volume of 9 μL.

### Generation of dsDNA Donor Template (HDR Template) *via* PCR Amplification or Plasmid Propagation

HDR templates containing site-specific HAs, a promoter gene, and GFP or Hu19 scFv-CD8α-CD28-CD3ζ CAR gene were amplified from corresponding pTwist Ampicillin high-copy plasmids (Twist Bioscience) using HA-specific PCR primers. CMV- (pEGFP-C1, Addgene), SCP-,^41^ CMVe-SCP-,^41,42^ PGK-,^43^ CMVmax- (pmaxGFP, Lonza), JET-,^44^ and EF-1α^45^-driven templates were amplified in a two-step PCR program: initial denaturation at 98 °C for 30 s, cycle denaturation at 98 °C for 10 s, and extension at 72 °C for 30 s per 1 kb amplicon for 40 cycles with a hold at 72 °C for 10 min. Each 50 μL PCR reaction contained a 10 ng amplification template (plasmid DNA), 0.5 μM of HA-specific forward and reverse primers, nuclease-free water (IDT), 3% DMSO, and 1× Phusion High-Fidelity PCR Master Mix with HF Buffer (Thermo Fisher Scientific). PCR products were purified using the NucleoSpin Gel and PCR Clean-up Kit (Macherey-Nagel) with double 20 μL elution. Plasmids containing CAG^45^-driven templates were propagated overnight in One Shot TOP10 Chemically Competent *E. coli* (Invitrogen, Thermo Fisher Scientific), and plasmid DNA was isolated and purified using anion exchange columns (HiSpeed Plasmid Maxi Prep, Qiagen). HDR templates were cut from the plasmid using restriction digestion, followed by purification on and isolation from agarose gel, and finally purified using NucleoSpin Gel (Macherey-Nagel). Purified HDR templates were collected and concentration measured on a NanoDrop One Microvolume UV–Vis Spectrophotometer (Thermo Fisher Scientific). Templates were up-concentrated using Amicon Ultra-0.5 mL 30 K centrifugal filters: 100 μg DNA per unit was transferred, filled with nuclease-free water to 500 μL, and centrifuged at 10,000 × g for 10 min to reduce the volume to 50 μL. DNA was washed twice with nuclease-free water and recovered into a fresh tube by unit inversion and centrifugation at 10,000 × g for 15 s. HDR templates were collected and diluted and concentrations quantified using Qubit dsDNA HS Assay Kit (Thermo Fisher Scientific). HDR donor templates of 0.5–1.5 μg reaction^–1^ were used for cellular studies, unless otherwise stated.

### Cell Line Transfection

A Lonza 4D-Nucleofector with Shuttle unit (V4SC-2960 Nucleocuvette Strips) was used for transfection, following the manufacturer’s instructions. For transfection, cells were harvested by centrifugation (200 × g, RT, 5 min) and re-suspended at 10 × 10^6^ cells mL^–1^ (2 × 10^5^ cells 20 μL^–1^) in the SF Cell Line Nucleofector X Kit buffer (Lonza), unless stated otherwise. The cell suspension was mixed with the RNPs, immediately transferred to the nucleocuvette, and transfected using the CA-137 Nucleofector program, except where indicated otherwise. After transfection, the cells were immediately re-suspended in the pre-warmed cultivation medium and plated onto 96-well, flat-bottom, non-treated plates (Falcon) and cultured at 37 °C in 5% CO_2_ incubators and maintained at a density of 0.5–1.0 × 10^6^ cells mL^–1^. After 48 h, the cells were harvested for the viability assay and genomic DNA, as described below. For the HDR template insertion, the HDR template was added to the cells and the suspension transferred to the RNPs immediately before transfection. The transfection parameters, cell recovery step, and proliferation conditions were as described above. The cells were harvested 48 h post-transfection for the viability assessment and after 7, 14, and 21 days for GFP insertion efficiency.

### Primary T-Cell Transfection

Forty-eight hours after isolation, the cells were harvested by centrifugation (300 × g, RT, 5 min) and re-suspended at 50 × 10^6^ cells mL^–1^ (1 × 10^6^ cells 20 μL^–1^) in the supplemented P3 Primary Cell Nucleofector Kit buffer (Lonza). The cells were mixed with HDRT, and the suspension was transferred to the RNPs immediately before transfection (Nucleofection program EH-115). After transfection, 80 μL of pre-warmed cultivation medium without IL-2 was added to the electroporation cuvettes. In the experiments with M3814 (Selleckchem), 80 μL of pre-warmed cultivation medium containing 2 μM of M3814 final concentration without IL-2 was added to the electroporation cuvettes. After a 10 min incubation at 37 °C, cells were transferred onto 96-well, flat-bottom, non-treated plates (Falcon) containing a pre-warmed cultivation medium pretreated with 2 μM of M3814 final concentration and 12.5 ng mL^–1^ of IL-2. The cells were seeded at a density of 2.5 × 10^6^ cells mL^–1^, or 1.3 × 10^6^ cells mL^–1^ in the experiments with M3814, and kept at 37 °C in 5% CO_2_ incubators. The viability assay was carried out 24 h post-transfection, after which the cells were reseeded in the fresh cultivation medium containing 12.5 ng mL^–1^ of IL-2. The insertion efficiency of CAR was measured after 7 days, and 11 or 13 days post-transfection.

### Flow Cytometry

Flow cytometric assessments were carried out on a CytoFLEX S instrument (Beckman Coulter) using a 96-well plate format. Measurements of cell viability, exhaustion marker expression, GFP expression, and CAR expression were performed on 10,000 or 20,000 single-cell events in Jurkat or T cells, respectively.

For the cell viability and GFP knock-in measurements, approximately 250,000 cells per sample were transferred onto 96-well V-bottom cell culture plates and assessed following a series of consecutive washing and staining steps. The first step included centrifuging the cells at 300 × g for 5 min at RT, discarding the supernatant, and washing Jurkat cells or T cells in 150 μL Dulbecco’s PBS/2% FBS (STEMCELL Technologies) or Cell Staining Buffer (BioLegend), respectively, followed by the second centrifugation and removal of supernatant. The final step included viability staining of Jurkat cells or T cells using 150 μL Dulbecco’s PBS/2% FBS with 7-amino-actinomycin D (7-AAD, 1:1000; Thermo Fisher) or 50 μL Cell Staining Buffer with Zombie Violet dye (1:200; BioLegend), respectively. The fluorescence excitation of 7-AAD is at 561 nm (yellow-green laser) of Zombie Violet at 405 nm (violet laser) and of GFP at 488 nm (blue laser).

For detection of CAR or exhaustion marker expression in primary T cells, approximately 250,000 cells per sample were transferred onto 96-well V-bottom plates, centrifuged at 300 × g for 5 min at RT, and re-suspended in 50 μL Cell Staining Buffer (BioLegend) with either PE Anti-Myc tag antibody [9E10] (1:30; Abcam) and Zombie Violet dye (1:200; BioLegend) for 30 min for CAR expression and viability or PD-1 Antibody (J116) [PE/Cy5.5] (1:200; Novus Biologicals), CD152 (CTLA-4) Monoclonal Antibody (14D3), PE-eFluor 610 (1:40; eBioscience), TIGIT Monoclonal Antibody (MBSA43), APC, (1:40; eBioscience), CD223 (LAG-3) Monoclonal Antibody (3DS223H) or eFluor 506 (1:60; eBioscience), and Zombie Violet dye (1:200; BioLegend) for 30 min for exhaustion marker expression and viability. Afterward, the cells were washed in two subsequent washing steps using 150 μL Cell Staining Buffer and finally re-suspended in 100 μL Cell Staining Buffer for the flow cytometry measurements. See Figure S9 for the gating strategy.

For detection of PDCD1 knockout efficiency, approximately 250,000 Jurkat cells per sample were transferred onto 96-well V-bottom plates, centrifuged at 300 × g for 5 min at 4 °C, re-suspended in 100 μL Cell Staining Buffer (BioLegend) with APC/Cyanine7 anti-human CD279 (PD-1) Antibody (1:100; BioLegend), and incubated for 30 min at 4 °C in the dark. The next step included two repeats of centrifugation at 300 × g for 5 min at 4 °C, supernatant removal, and cell washing in 150 μL ice-cold Cell Staining Buffer (BioLegend). In the final step, the cells were re-suspended in 100 μL Cell Staining Buffer for the flow cytometry measurements using red laser (638 nm).

### Genomic DNA Extraction and PCR Amplification

Cells were harvested 48 h post-transfection by centrifugation (1000 × g, 10 min) in 96-well, V-bottom plates (Greiner), washed with PBS (Sigma Aldrich), and lysed in 20 μL QuickExtract DNA Extraction Solution (Epicenter, Lucigen). DNA was extracted following the manufacturer’s protocol of 15 min at 65 °C, 15 min at 68 °C, and 10 min at 95 °C, cooled down, and stored at 4 °C. Genomic DNA was diluted 20× in nuclease-free water before amplicon PCR reactions.

### Targeted Amplicon Sequencing

Extracted genomic DNA was quantified using the NanoDrop (Thermo Fisher Scientific). Amplicons were constructed in two PCR steps: in the first PCR, regions of interest (150–400 bp) were amplified from 10 to 30 ng of genomic DNA with primers containing Illumina forward and reverse adapters on both ends, using Phusion High-Fidelity PCR Master Mix (Thermo Fisher Scientific) supplemented with 0.4 U Phusion Hot Start II High-Fidelity PCR Master Mix (Thermo Fisher Scientific) per reaction. Amplification products were purified with Agencourt AMPure XP beads (Ramcon), using the sample-to-bead ratio of 1:1.8. The DNA was eluted from the beads with nuclease-free water and the size of the purified amplicons analyzed on a 2% agarose E-gel using the E-gel electrophoresis system (Thermo Fisher Scientific). In the second PCR, unique pairs of Illumina-compatible indexes (Nextera XT Index Kit v2) were added to the amplicons using the KAPA HiFi HotStart Ready Mix (Roche). The amplified products were purified with Agencourt AMPure XP beads (Ramcon), using the sample-to-bead ratio of 1:1.8. The DNA was eluted from the beads with 10 mM Tris–HCl pH = 8.5 + 0.1% Tween 20. Sizes of the purified DNA fragments were validated on a 2% agarose gel using the E-gel electrophoresis system (Thermo Fisher Scientific), quantified using Qubit dsDNA HS Assay Kit (Thermo Fisher), and then pooled in equimolar concentrations. The quality of the amplicon library was validated using the Bioanalyzer High-Sensitivity DNA Kit (Agilent) before sequencing. The final library was sequenced on the Illumina MiSeq System using the MiSeq Reagent Kit v.2 (300 cycles, 2 × 250 bp, paired-end reads). De-multiplexed FASTQ files were obtained from BaseSpace (Illumina).

### NGS Data Analysis

An initial quality assessment of the obtained reads was performed with FastQC36. The sequencing data were aligned and analyzed with the CRISPResso2 software,^26^ using the CRISPRessoBatch command with the parameters *--cleavage_offset 1 --quantification_window_size 10 --quantification_window_center 1 --expand_ambiguous_alignments* for the INDEL frequency analysis. For the ORF disruption analysis, the CRISPRessoBatch command with the parameters *--cleavage_offset 1 −coding_seq < EXON_SEQ > −-quantification_window_size 0 --quantification_window_center 1 --expand_ambiguous_alignments* was used. Modification rates from the CRISPResso2 software output were analyzed in MS Excel.

### Statistical Analysis

Analysis was performed in RStudio, using either two-tailed *T*-test or Fisher’s exact test. The level of significance used was 0.05.

## Supporting Information Available

On-target and off-target spacer and PAM sequences (XLSX)
Engineering of MAD7; optimization of in cellulo editing activity; sequence logos of analyzed crRNAs; frequency of ribonucleotide bases; correlation between crRNA screen and crRNA validation experiments; surface expression of PDCD1 protein; editing efficiency of crAAVS1; HDRT insertion efficiency at AAVS1 using various promoters; identification of optimal PGA amount; identification of optimal RNP and HDRT amount and ratio; gating strategy chart of flow cytometry data; on-target crRNA, PAM, and primer sequences; and off-target crRNA, PAM, and primer sequences (PDF)

## Abbreviations

CAR: chimeric antigen receptor
Cas12a: CRISPR-associated protein 12a (previously known as Cpf1)
Cas9: CRISPR-associated protein 9
CD247: cluster of differentiation 247 or T-cell receptor T3 zeta chain
CRISPR: clustered regularly interspaced short palindromic repeats
crRNA: CRISPR RNA
CTLA4: cytotoxic T-lymphocyte-associated protein 4
DNA-PK: DNA-dependent protein kinase
DNMT1: DNA (cytosine-5)-methyltransferase 1
HDR: homology-directed repair
LAG3: lymphocyte-activation protein 3
NHEJ: non-homologous end joining
NLS: nuclear localization signal
PAM: protospacer adjacent motif
PDCD1: programmed cell death protein 1
PGA: poly-l-glutamic acid
PTPN11: protein tyrosine phosphatase non-receptor type 11 (SHP-2)
PTPN6: protein tyrosine phosphatase non-receptor type 6 (SHP-1)
RNP: ribonucleoprotein
TCR: T-cell receptor
TIGIT: T-cell immunoreceptor with Ig and ITIM domains
TIM3: T-cell immunoglobulin and mucin domain-containing protein 3
TRAC: T-cell receptor alpha constant
tracrRNA: trans-activating CRISPR RNA
TTTV: TTT[A/G/C]
YTTN: [T/C]TT[A/T/G/C]
