# Supplementary material for: The CRISPR-Cas12a Platform for Accurate Genome Editing, Gene Disruption, and Efficient Transgene Integration in Human Immune Cells
Source: ACS Synth Biol. 2023 Feb 7;12(2):375–89. doi: 10.1021/acssynbio.2c00179 (PMC9942205; doi:10.1021/acssynbio.2c00179)
Supplement: Supplementary file 2 — sb2c00179_si_002.pdf [file sb2c00179_si_002.pdf]

Supporting Information

CRISPR-Cas12a Platform for Accurate Genome Editing, Gene Disruption, and Efficient Transgene Integration in Human Immune Cells

Marina Mohr[1], Nkerorema Djodji Damas[1], Johanne Gudmand-Høyer[1], Katrine Zeeberg[1], Dominika Joanna Jedrzejczyk[1], Arsenios Vlassis[1], Martí Morera-Gómez[1], Sara Pereira-Schoning[2], Urška Puš[1], Anna Oliver-Almirall[1], Tanja Lyholm Jensen[1], Roland Franz Baumgartner[2], Brian Tate Weinert[1], Ryan T. Gill[1,2*], Tanya Warnecke[2*]

[1]Novo Nordisk Foundation Center for Biosustainability, Technical University of Denmark, Kemitorvet 220, 2800 Kongens Lyngby, Denmark

[2]Artisan Bio, 363 Centennial Parkway, Suite 310, Louisville, CO 80027

[*] e-mail: rtg@biosustain.dtu.dk, tanya@artisancells.com

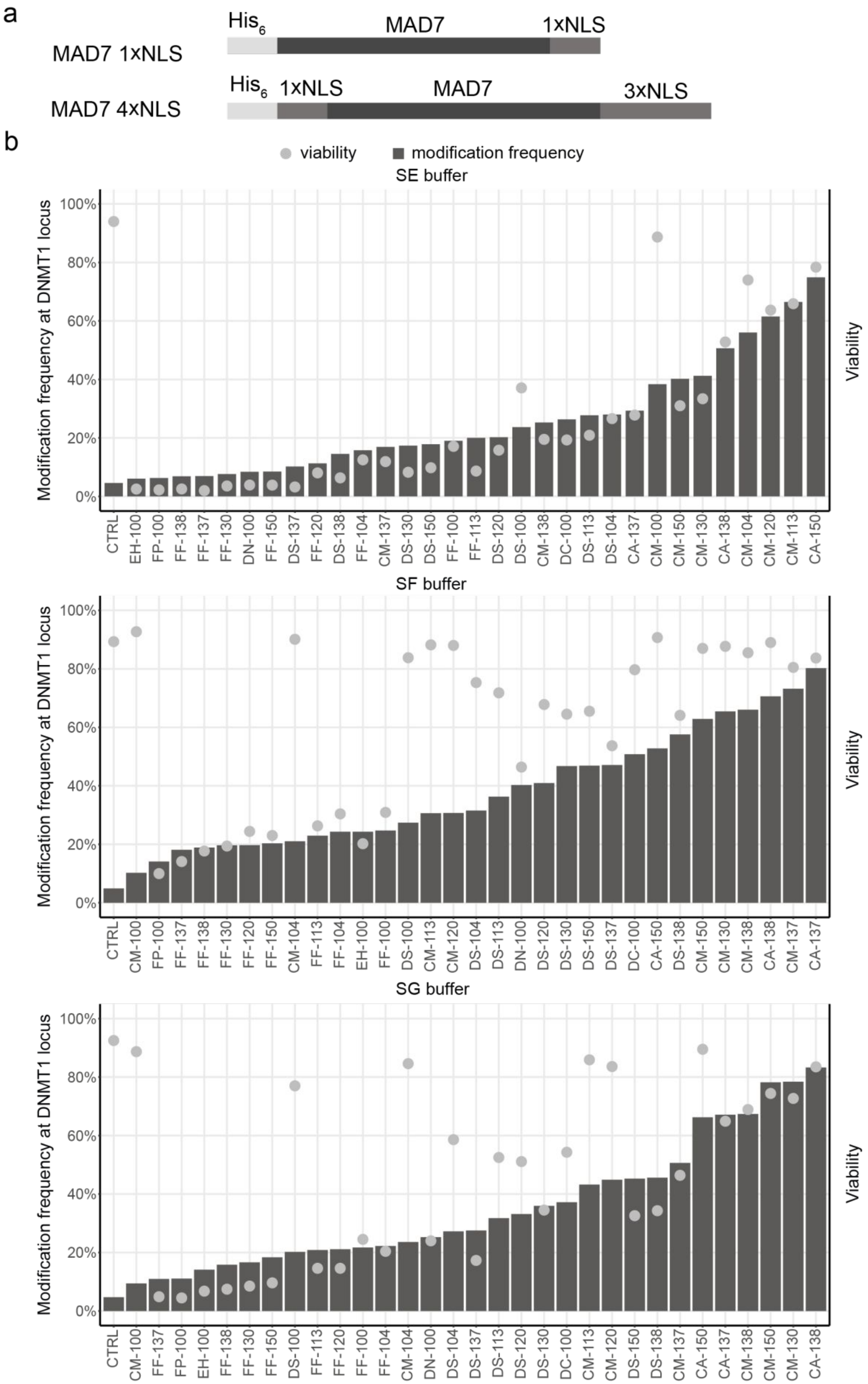
a
His6
MAD7
1xNLS
MAD7 1xNLS
His6
1xNLS
MAD7
3xNLS
MAD7 4xNLS
b
viability
modification frequency
SE buffer
Modification frequency at DNMT1 locus
Viability
100%
80%
60%
40%
20%
0%
CTRL EH-100 FP-100 FF-138 FF-137 FF-130 DN-100 FF-150 DS-137 FF-120 DS-138 FF-104 CM-137 DS-130 DS-150 FF-100 FF-113 DS-120 DS-100 CM-138 DC-100 DS-113 DS-104 CA-137 CM-100 CM-150 CM-130 CA-138 CM-104 CM-120 CM-113 CA-150
SF buffer
Modification frequency at DNMT1 locus
Viability
100%
80%
60%
40%
20%
0%
CTRL CM-100 FP-100 FF-137 FF-138 FF-130 FF-120 FF-150 CM-104 FF-113 FF-104 EH-100 FF-100 DS-100 CM-113 CM-120 DS-104 DS-113 DN-100 DS-120 DS-130 DS-150 DS-137 DC-100 CA-150 DS-138 CM-150 CM-130 CM-138 CA-138 CM-137 CA-137
SG buffer
Modification frequency at DNMT1 locus
Viability
100%
80%
60%
40%
20%
0%
CTRL CM-100 FF-137 FP-100 EH-100 FF-138 FF-130 FF-150 DS-100 FF-113 FF-120 FF-100 FF-104 CM-104 DN-100 DS-104 DS-137 DS-113 DS-120 DS-130 DC-100 CM-113 CM-120 DS-150 DS-138 CM-137 CA-150 CA-137 CM-138 CM-150 CM-130 CA-138

12

Figure S1:

(a) Engineering of MAD7. Diagram of MAD7 showing position and identity of nuclear localization signal(s) (NLS).

(b) Optimization of *in-cellulo* editing activity. Modification frequency at the DNMT1 locus (bars; t=1) and cell viability (circles; t=1) of Jurkat cells as a function of Lonza nucleofection buffers (SE, top; SF, middle; SG, bottom) and various programs. CTRL is UNMOCK in their respective buffers.

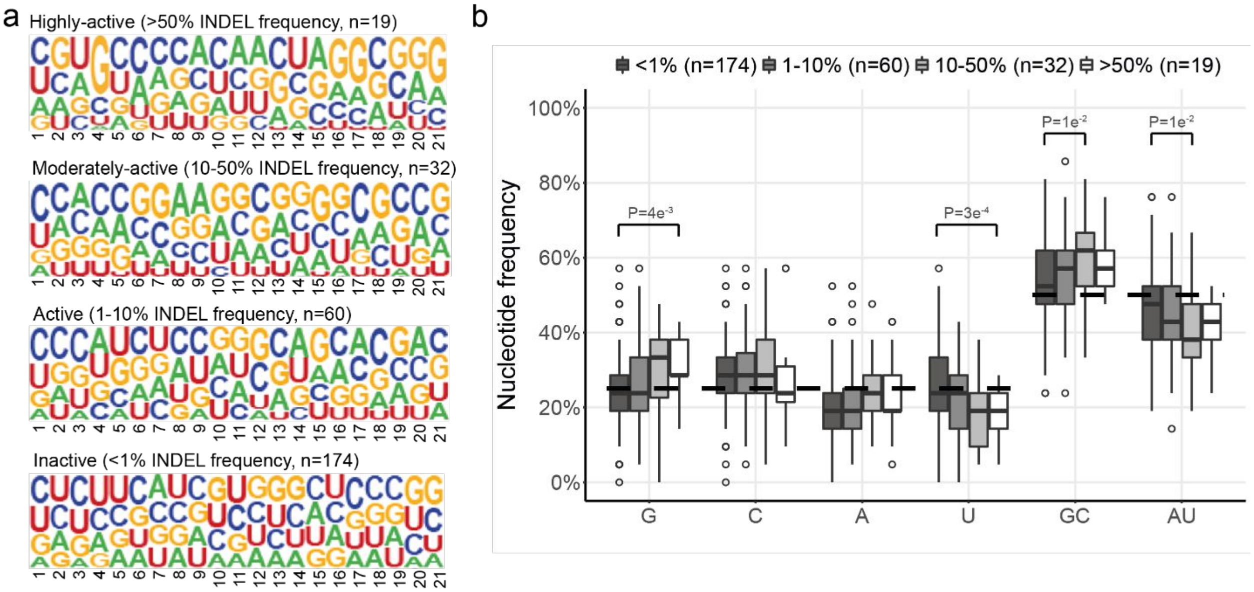

Figure S2:

(a) Sequence logos of analyzed crRNAs. Sequence logos comparing DNA-complementary crRNA sequences of highly-active (≥50% INDELs), moderately-active (10-50% INDELs), active (1-10% INDELs), and inactive (≤1% INDELs) crRNAs show no strong biases for ribonucleotides at specific positions, however, guanine appeared overrepresented and uracil underrepresented on highly-active and moderately-active crRNAs.

(b) Frequency of ribonucleotide bases. Nucleotide frequency on inactive (≤1% INDELs; dark grey box), active (1-10% INDELs; medium grey box), moderately-active (10-50% INDELs; light grey box), and highly-active (≥50% INDELs; white box) crRNAs, with significant enrichment of guanine and depletion of uracil on highly-active crRNAs compared to inactive crRNAs (Fisher's exact test, $P=4\times10^{-3}$ and $P=3\times10^{-4}$, respectively). Also, significant enrichment of guanine-cytosine content and depletion of adenine-uracil content was observed on moderately-active crRNAs compared to inactive crRNAs (Fisher's exact test, $P=1\times10^{-2}$).

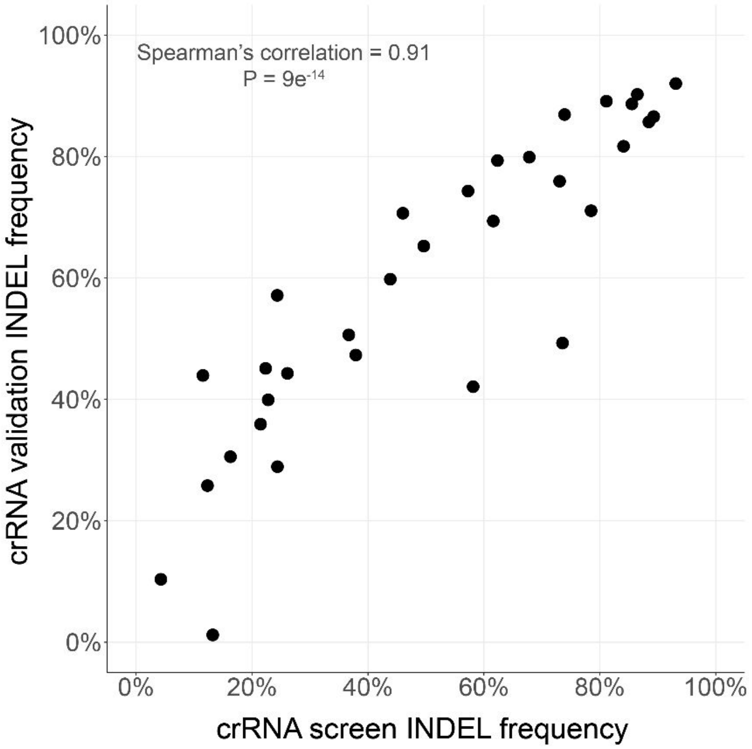

Figure S3: Correlation between crRNA screen and crRNA validation experiments. INDEL frequency in the crRNA validation experiment versus INDEL formation in the crRNA screen experiment show significant correlation of the measurements (Spearman's correlation = 0.91; $P=9\times10^{-14}$), highlighting reproducibility of the INDEL assay.

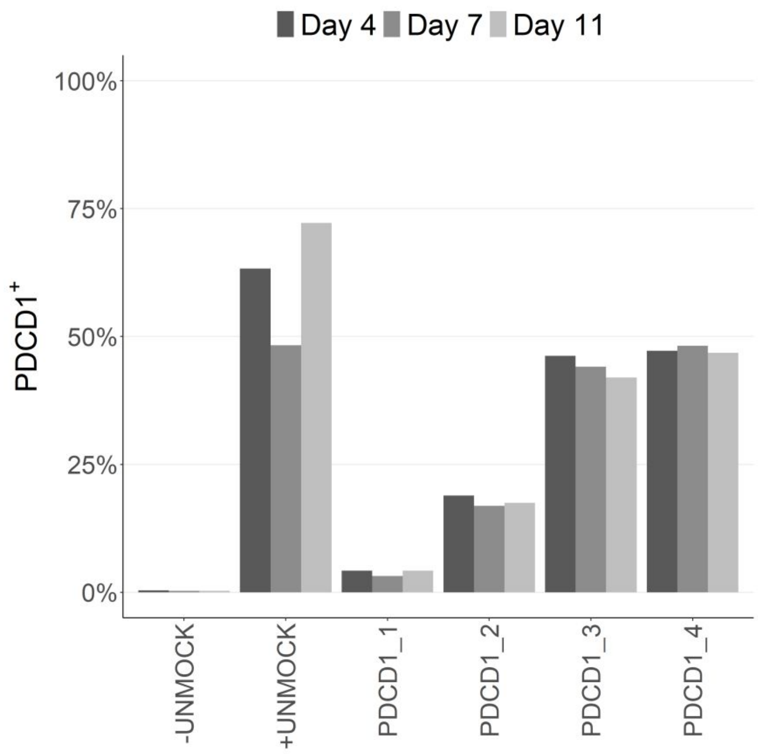

Figure S4: Surface expression of PDCD1 protein. Normalized surface expression of PDCD1 protein in Jurkat cells after transfection with four highly-active crRNAs PDCD1_2, PDCD1_3, PDCD1_4, and PDCD1_5 (see Fig. 2) measured at days 4 (dark grey), 7 (grey), and 11 (light grey) post-transfection. –UNMOCK is unmodified and unstained control, and +UNMOCK is unmodified and stained control. It is important to note that crPDCD1_1 targets the PDCD1 gene at the extracellular domain of the protein, while crPDCD1_2, and PDCD1_3 and PDCD1_4 target the transmembrane domain and the cytoplasmic domain of the protein, respectively.

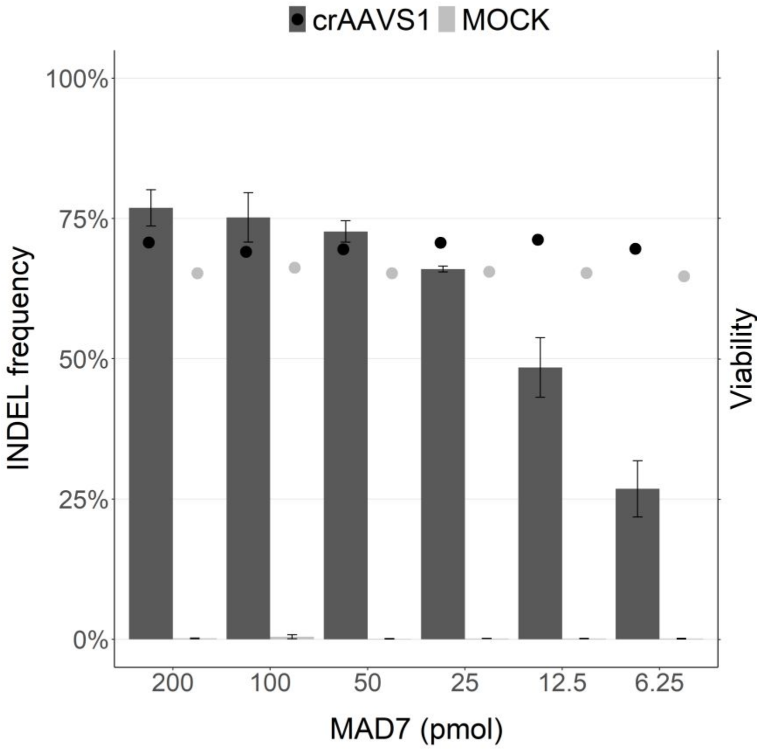

Figure S5: Editing efficiency of crAAVS1. INDEL frequency at the AAVS1 locus (t=3; Mean ± SD) and cell viability (t=3; Mean) of Jurkat cells measured at day 2 post-transfection as a function of MAD7-RNP amounts (pmol; constant ratio of 2:3 MAD7:crRNA) for improved *in-cellulo* editing. Modifications were achieved using the best performing Lonza nucleofection program SF-CA-137 (see Fig. 1B). Dark grey and light grey bars and circles represent mean INDEL frequency and cell viability (in that order) in treated and MOCK (crIDTneg1) samples, respectively.

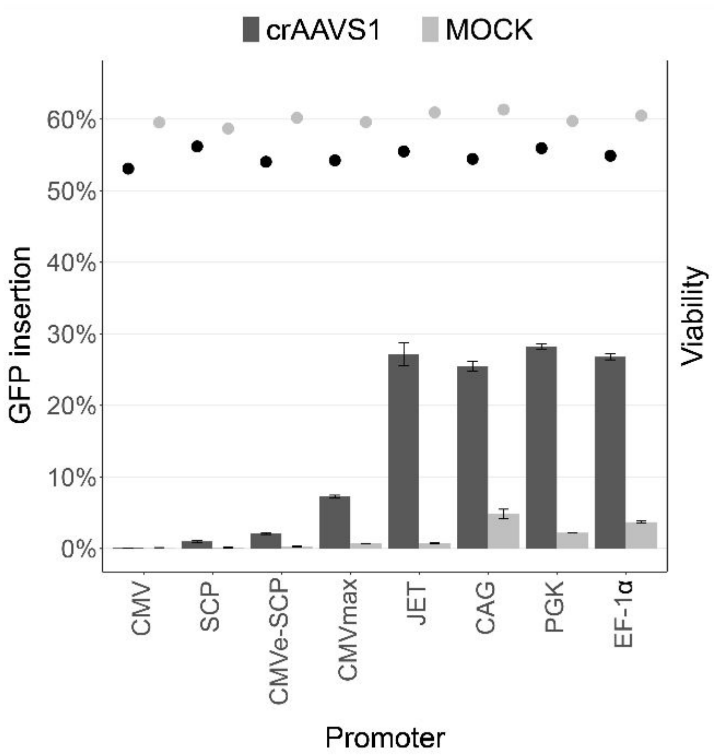

Figure S6: HDRT insertion efficiency at AAVS1 using various promoters. GFP insertion efficiency at AAVS1 (t=3; Mean ± SD) and cell viability (t=3; Mean) of Jurkat cells measured at day 14 and day 2 post-transfection, respectively. HDR templates consisting of eight different promoters and flanked with symmetric homology arms of 500 bp in the amount of 0.5-µg reaction^{-1} were used. Size of promoters in base pairs: CMV, 508; SCP, 81; CMVe-SCP, 385; CMVmax, 947; JET, 195; CAG, 1723; PGK, 511; EF-1α, 1195. Dark grey and light grey bars and circles represent mean insertion frequency and cell viability (in that order) in treated and MOCK (crIDTneg1) samples, respectively.

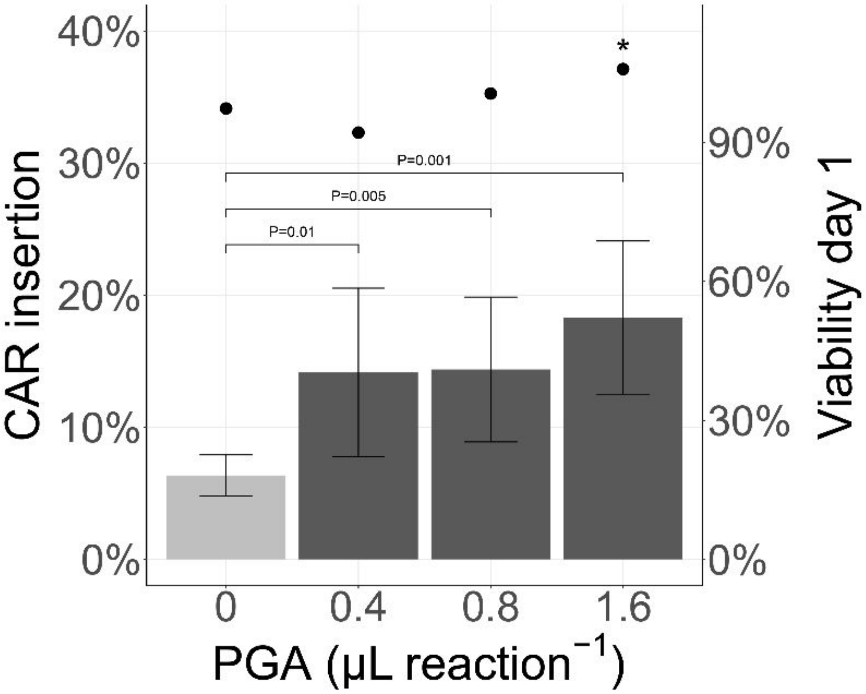

Figure S7: Identification of optimal PGA amount. CAR insertion efficiency at AAVS1 (N=2; t=3; Mean ± SD) in primary Pan T-cells as a function of poly-L-glutamic acid (PGA; µL reaction^{-1}) measured at day 7 post-transfection. Cell viability (N=2; t=3; Mean) was measured 24-h post-transfection, and normalized cell viability (relative to UNMOCK) plotted. Amount of HDRT and MAD7-RNP was 1-µg and 100:150 pmol, respectively. Nucleofection program P3-EH-115 for transfection of primary T-cells was used. *N* represents number of biological replicas, and *t* number of technical replicas per *N*. CAR insertion frequency was significantly higher in the samples treated with PGA compared to the untreated sample (two-tailed T-test, P=0.01, P=0.005, and P=0.001, in that order). Cell viability was significantly higher in the treatment with 1.6-µL PGA reaction^{-1} compared to the untreated sample (asterisk; two-tailed T-test, P=9e^{-4}).

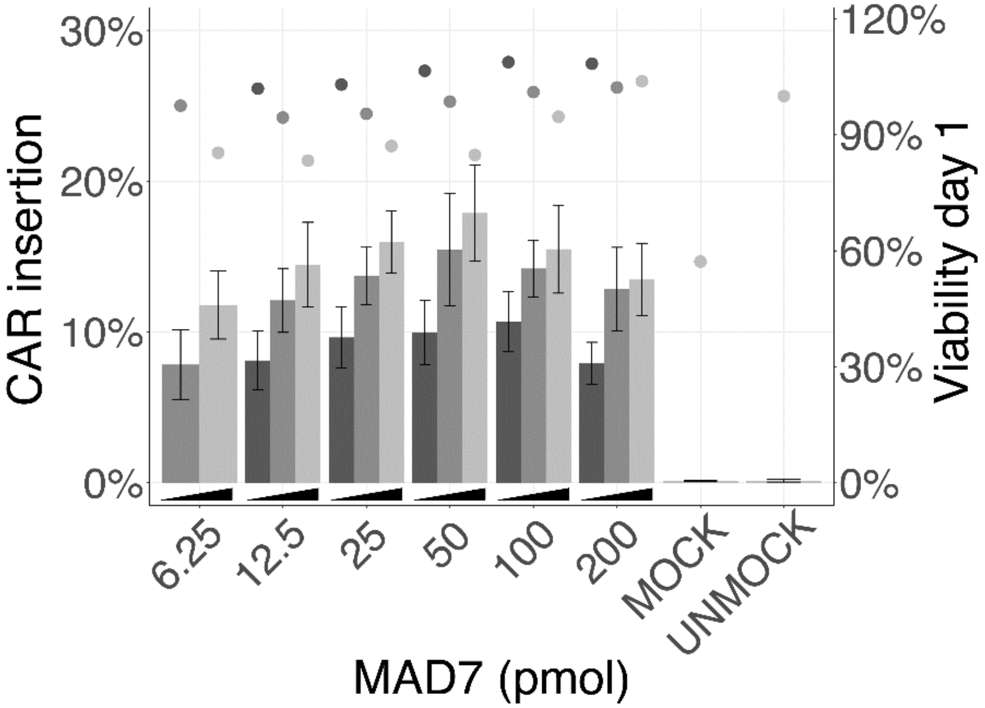

Figure S8: Identification of optimal amount and ratio of RNP and HDRT. CAR insertion efficiency at AAVS1 (N=2; t=3; Mean ± SD) in primary Pan T-cells measured at day 7 post-transfection as a function of RNP (pmol; constant ratio of 2:3 MAD7:crRNA) and HDR template (µg) amounts. Cell viability (N=2; t=3; Mean) was measured 24-h post-transfection, and normalized cell viability (relative to UNMOCK) plotted. Amount of HDRT, represented by the gradient below the bars, increases from 0.5- (dark grey), 1- (grey), to 1.5-µg (light grey). Nucleofection program P3-EH-115 for transfection of primary T-cells was used. *N* represents number of biological replicas, and *t* number of technical replicas per *N*.

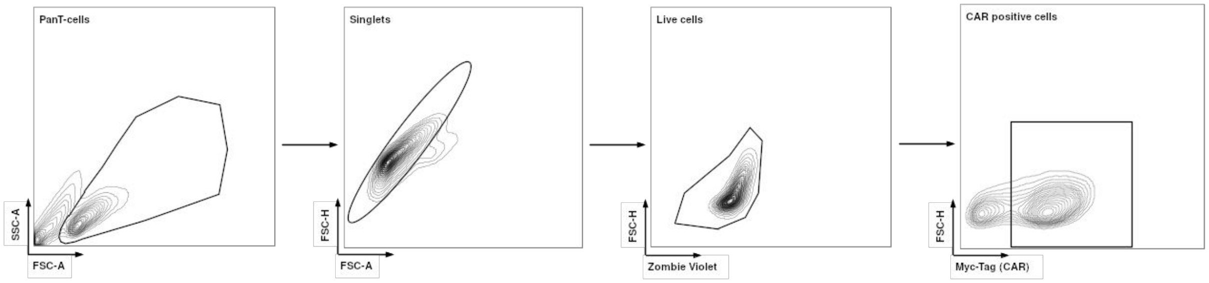

Figure S9: Gating strategy chart of flow cytometry data. From left to right: Pan T-cells gate defined from FSC-A vs. SSC-A; singlets gate defined on FSC-A vs. FSC-H; live cells gate defined using FSC-H and a viability dye. Further gating was made in accordance with the used fluorophore vs. FSC-H, and gated to control samples of the respective experiment.

TableS1: On-target and off-target spacer and PAM sequences. 'On-target' displays 298 unique crRNA (spacer) sequences targeting the following genes: immune checkpoint receptors PDCD1, TIM3, LAG3, TIGIT, and CTLA4, checkpoint phosphatases PTPN6 (SHP-1) and PTPN11 (SHP-2), and TCR signaling subunit CD247 (CD3ζ); as well as the sequence of crRNA targeting AAVS1 safe-harbor-site. Moreover, PAM sequences together with forward and reverse primer sequences for amplification are displayed. 'Off-target' displays alternative crRNA (spacer) sequences, number of mismatches between crRNA and target DNA sequence, and PAM sequences, as well as forward and reverse primer sequences for amplification.
